# Light-induced changes of the subretinal space of the temporal retina observed via optical coherence tomography

**DOI:** 10.1038/s41598-019-50057-8

**Published:** 2019-09-20

**Authors:** Alina Messner, René M. Werkmeister, Gerald Seidel, Hannes Stegmann, Leopold Schmetterer, Valentin Aranha dos Santos

**Affiliations:** 1 Center for Medical Physics and Biomedical Engineering, Medical University of Vienna, 1090 Vienna, Austria; 2 Department of Ophthalmology, Medical University of Graz, 8036 Graz, Austria; 3 Department of Clinical Pharmacology, Medical University of Vienna, 1090 Vienna, Austria; 4 Singapore Eye Research Institute, The Academia, Singapore, 169856 Singapore; 5 Lee Kong Chian School of Medicine, Nanyang Technological University, Singapore, 636921 Singapore; 6 Christian Doppler Laboratory for Ocular and Dermal Effects of Thiomers, Medical University of Vienna, 1090 Vienna, Austria

**Keywords:** Retina, Imaging and sensing

## Abstract

Photoreceptor function is impaired in many retinal diseases like age-related macular degeneration. Currently, assessment of the photoreceptor function for the early diagnosis and monitoring of these diseases is either subjective, as in visual field testing, requires contact with the eye, like in electroretinography, or relies on research prototypes with acquisition speeds unattained by conventional imaging systems. We developed an objective, noncontact method to monitor photoreceptor function using a standard optical coherence tomography system. This method can be used with various white light sources for stimulation. The technique was applied in five volunteers and detected a decrease of volume of the subretinal space associated with light adaptation processes of the retina.

## Introduction

Optophysiology refers to the measurement of physiological functions with optical methods^1,2^. It aims to quantify *intrinsic optical signals*, a collective term for all stimulus-evoked changes of optical properties in a tissue, including scattering intensity, optical path length, polarisation and absorption^3^.

Intrinsic optical signals have been proposed as a biomarker for photoreceptor function^4,5^. Functional deterioration might be detectable earlier than structural changes and precede clinical signs in retinal diseases^6,7^. Therefore, imaging of intrinsic optical signals could enable early detection of retinal disorders and improve monitoring of their therapy.

Optical coherence tomography (OCT) has revolutionized ophthalmology by providing new insights into the morphology and function of the retina. The first successful measurements of retinal physiology using OCT were performed in isolated retinas^8,9^. Since then, intrinsic optical signal imaging has obtained compelling results in humans *in vivo*^4,10,11^.

Ophthalmologic studies targeting intrinsic optical signal imaging without utilization of OCT usually employ fundus photography or scanning laser ophthalmoscopy (SLO), thus acquiring two-dimensional images of the posterior pole^5–7,12^. While having the advantage of being very fast, these approaches lack detailed depth information^5,13^.

In early OCT based studies, the most frequently assessed type of intrinsic optical signals was the change in the scattering properties of the inner segment/outer segment (IS/OS) junction and the photoreceptor outer segments^9,13–16^. Recently, changes in optical path length of the outer retinal layers have been increasingly reported^4,10,17–19^.

The measurement of intrinsic optical signals via intensity/amplitude changes faces some challenges that are difficult to overcome. Mixed positive and negative intensity changes are contributing to the light induced responses, complicating both the evaluation and interpretation^3^. In spectral domain OCT, the sensitivity *roll-off* causes the OCT amplitude to change in case of an axial shift of the observed structure^20^. Furthermore, in cone photoreceptors, the *Stiles-Crawford Effect* leads to intensity changes depending on the incidence angle of the impinging light^21^. These factors make intrinsic optical signals imaging based on intensity data highly susceptible to artefacts by even small motion^3,15,21^. Hence, synchronous intensity changes in all reflection bands of the retina may indicate a systematic error that might be caused by a varying coupling efficiency due to a changing angle of incidence.

Changes in optical path length can be either due to distance changes between the reflectors or due to alterations of the refractive index. Unlike the intensity of the OCT signal that provides information about changes in scattering and reflectivity, the optical path length is not influenced by depth and position and does thus not change with intensity.

Employment of the optical path length change for the detection of intrinsic optical signals has already provided some reliable results in animals and humans *in vivo*^10,11,17^. Zhang *et al*. found an increase in optical path length between IS/OS junction and retinal pigment epithelium (RPE) after light stimulation in mice^17^. Hillmann *et al*. presented fast expansion of outer segments after light stimulation in humans^10^. However, these measurements required the use of specialized and expensive custom-built high-speed or full-field OCT systems that are not available in the clinical practice.

Here we explore changes in the outer retina using a commercial OCT platform and advanced image processing before and after long white light stimulation. We report decreases in optical path length between IS/OS junction and RPE after light stimulation in the peripheral human retina *in vivo* using a commercially available OCT system. We conduct that these changes have their origin in a volume change of the subretinal space, between the photoreceptors and the RPE.

## Results and Discussion

### Optical path length decreases between IS/OS junction and RPE after light exposure

**Figure 1:**
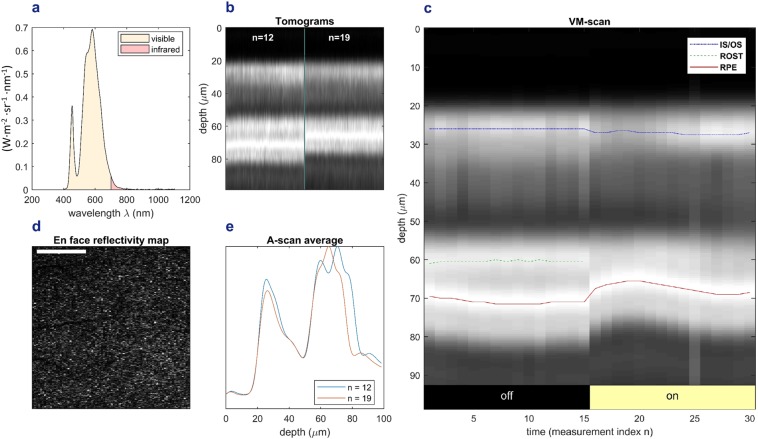
Imaging of the temporal, outer retina before and during the light stimulation period with white light in a healthy, young volunteer.”n” denotes the measurement index of one volume and therefore represents a non linear axis of time. Volumes are presented as their A-scan average, which is the average of all their A-scans. The duration of each measurement was about five seconds and a break of one minute was held between all measurements except 15 and 16 where the break was five minutes long, due to the longer initial light exposure. (**a**) Spectrum of the stimulation light. (**b**) Comparison of two measurements (number 12 and 19) before and during the light stimulation period. The OCT tomogram represents a section of the outer retina, containing photoreceptor outer segments and RPE, and has been averaged along the fast axis, which can be understood as one row of A-scans averaged into one column. (**c**) VM-scan: rod outer segment tips (ROST) and RPE are clearly separated before light exposure. After light exposure, a distinct change in optical path length between inner segment/outer segment (IS/OS) junction and RPE leads to the convergence and merging of the reflective bands of the ROST and the RPE. Each column represents the A-scan average of a whole volume at any distinct time point (measurement index). (**d**) En face map of the region of interest at the photoreceptor level. Scale bar corresponds to 3.5 degrees. Retinal vessels were used as landmarks in the en face maps to assure a constant measurement region. (**e**) Normalized scattering intensity over depth in two measurements (index 12 and 19). The merging of the reflective bands corresponding to the ROST and the RPE is observable in n = 19.

The temporal retina of a healthy young subject was imaged by means of a standard resolution OCT platform multiple times before and after white light stimulation with a light therapy device (Fig. 1). Stabilization of the measurement region during experiments was assured by software tracking and confirmed in the *en face* images after post-processing (ref. methods section). After the baseline measurements, light exposure took place before each measurement and lasted five minutes for the first stimulation and one minute for every subsequent one. For each volumetric measurement, all A-scans were registered to the IS/OS junction and averaged, yielding a single A-scan average of reflective bands representing one volume. The A-scan average was then cropped to the region of interest. These are presented over time, as a function of the measurement index. These volume motion (VM) scans (Fig. 1c) demonstrate the decrease in the optical path length between IS/OS junction and RPE during stimulation. The largest decrease of around six microns (44.7 µm at baseline vs. 38.7 µm during stimulation) was found after eight minutes of cumulative light exposure and led to a merging of the reflective bands of RPE and rod outer segment tips (ROST) (Fig. 1b,c,e).

**Table 1:** Measurement data for all subjects.

Subject	1	2	3	4	5
mean distance at baseline ± SD (μm)	44.7 ± 0.6	44.8 ± 0.2	38.9 ± 0.4	42.2 ± 0.7	42.9 ± 1.2
min. distance during stimulation (μm)	38.7	41.2	34.9	37.2	36.2
difference (μm)	6.0	3.6	4.0	5.1	6.6
time to min. distance (min)	8	9	9	10	10
mean dist. during stimulation ± SD (μm)	40.0 ± 1.1	41.9 ± 0.5	36.1 ± 0.7	38.6 ± 1.0	38.0 ± 1.2
p-value baseline vs. stimulation	<0.001	<0.001	<0.001	<0.001	<0.001

The path length changes between IS/OS junction and RPE are shown. The mean distances at the baseline and during the stimulation period are given with the standard deviation. The difference between the mean baseline distance and the minimum distance after light exposure is presented. “Time to min. distance” is the cumulative time of retinal light exposure that is required to reach the minimum distance. Bonferroni-corrected p-values are given for comparison of the mean distances at baseline vs. during the light stimulation period.

### The optical path length decrease occurs due to a volume change in the subretinal space

In order to investigate which structure is responsible for the observed change, the optical properties of the different measured tissues have to be considered. There are two anatomical compartments located between the reflective bands of the IS/OS junction and the RPE. The first is constituted by the outer segments of the photoreceptors, the other by the subretinal space, the extracellular space surrounding the photoreceptors and bordering on the apical surface of the RPE cells and filled with the interphotoreceptor matrix^22^. Before light stimulation, the reflective band of the ROST is clearly separated from the reflective band of the RPE. After light exposure, the optical path length between ROST and RPE decreases, causing the corresponding reflecting bands to merge. This can be observed by comparing both the tomograms before and during the light stimulation period (Fig. 1b) as well as the A-scan averages of these measurements (Fig. 1e). This finding demonstrates that a change in the subretinal space is responsible for the major part of the optical path length decrease between IS/OS junction and RPE. Since the band of the outer segment tips merges with the RPE, they either contribute less to the decrease or even increase in optical path length.

**Figure 2:**
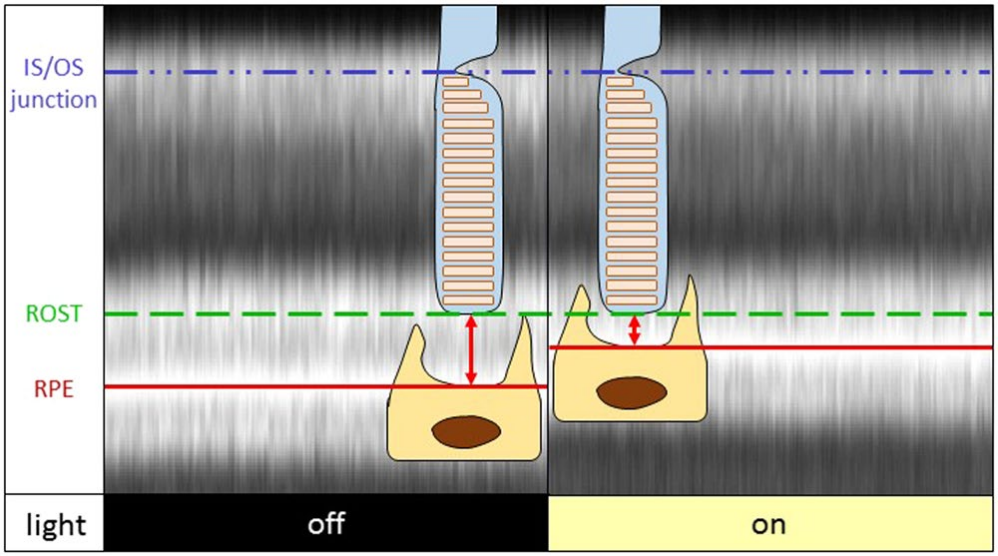
B-scans of the outer retina before (“off”, measurement index 12) and after (“on”, measurement index 19) light stimulation registered and flattened to the IS/OS junction and averaged along the fast axis. Schematic interpretation of the cells’ involvement in the path length decrease between IS/OS junction and RPE after light stimulation is presented as an overlay. In the baseline measurement (“off”), the reflectors of ROST and RPE are clearly separated, while after light stimulation (“on”) their peaks have merged. These findings show that the outer segment tips and RPE are drawn closer together after light exposure. This is associated with a decrease in volume of the subretinal space, as indicated by the red arrows.

Optical path length OPL is the product of the geometric distance d and the refractive index of the medium n (OPL = d ∙ n). Consequently, either the distance between ROST and RPE becomes shorter after light stimulation and, thus, the volume of the subretinal space smaller, or the refractive index of the subretinal space decreases. The refractive index of water is approximately 1.33^23^, which we assume as the lower bound of possible refractive indices of the subretinal space *after* light stimulation. To achieve the observed decrease in optical path length of 13.5% via a decrease in refractive index, the refractive index before light stimulation would have to be 1.54, similar to the one of pure sodium chloride (NaCl)^24^. The reported refractive index of the outer photoreceptor segments is 1.408 in rods^25^. Since the subretinal space is a type of extracellular space^26^, we assume the refractive index to be lower than that of the outer segments. In OCT, for a reflection to occur, the refractive indices of two bordering media have to be different. Therefore, 1.408 is the upper bound for possible refractive indices of the subretinal space in baseline conditions. Otherwise, the reflective band at the outer segment tips would not be formed. Consequently, the optical path length change, potentially caused by a shift in refractive index, constitutes at most 38% of the measured change. Therefore, the difference in geometric distance has to account for at least 62% of the reported change. A graphical visualization of the process is depicted in Fig. 2. To further investigate this hypothesis in the future, experimental information about the optical properties could be gained by using the method proposed by Almasian *et al*. on smaller shifts induced by a lower exposure to the stimulation light^27^.

### Influence of the stimulus spectrum on the measurement results

In an effort to investigate the influence of the spectrum used for light stimulation, the measurement series was repeated in the same subject using a fundus camera that provided the same luminance as the previous device but emitted a spectrum with a larger proportion in the infrared region and, thus, a different radiance. The observed changes were similar to the first measurement series, but the maximum change in length was smaller and amounted to 3.73 μm (8.7%).

### Thermal effects do not contribute to the change

Further, the possible influence of thermal effects on both, the refractive index and volume of compartments, was considered. A theoretical temperature increase from e.g. 35 to 42 degrees Celsius would decrease the refractive index by only about 8.7 ∙ 10^−4^, (1.2 ∙ 10^−4^ per degree Celsius), if the compartment would consist mainly of water^23^, and by about 2.0 ∙ 10^−2^ (2.8 ∙ 10^−3^ per degree Celsius)^28^ if the compartment would consist mainly of phospholipids, both of which are negligible. The volume change due to heating, which would have the opposite effect, namely an increase in path length, can also be neglected, since water is nearly incompressible.

To demonstrate the independence of the occurrence of the effect, from certain light source spectra and other characterizing features, like the fixation target or sitting position, two different white light sources were employed for the current study. Stimulating in between measurements has the disadvantage that immediate responses of the retina cannot be observed. However, there are two advantages: using a different optical path for stimulation and measurement avoids introducing uncertainties by heating the optical parts along the way, which could alter measurement results^29^. In addition, the simplified measurement protocol facilitates easy implementation into standard imaging procedures.

### Repetition of measurements in other subjects

**Figure 3:**
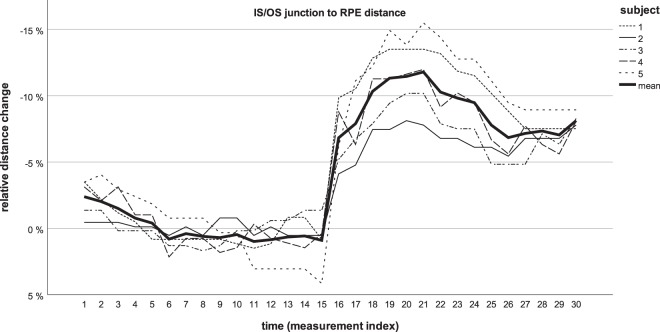
Mean relative change of the distance between IS/OS junction and RPE. The mean and individual data of five subjects is given relative to the mean baseline distance (measurements 1 to 15). The time axis represents the measurement index and is therefore not linear. Before measurement 16, the retina was stimulated with white light for five minutes, for each following measurement the illumination lasted one minute. The variance of the baseline measurements was similar among the subjects.

The measurement protocol was completed for four additional young, healthy subjects and confirmed our previous findings. The time course of the mean distance between IS/OS junction and RPE across all five subjects (three female, two male; mean age of 28 ± 2 years) can be seen in Fig. 3. In Table 1, the data for the individual subjects using white light stimulation with the light therapy device are given. Under ambient light conditions, in all subjects the mean distance between IS/OS junction and RPE in the measured area was 43.0 μm ± 2.4 μm. After light stimulation, this distance decreased to 37.6 μm ± 2.4 μm, corresponding to a mean maximum decrease of 5.1 μm ± 1.3 μm (12%). The mean cumulative light exposure time after which the minimum distance was first reached was 9.2 ± 0.8 minutes. Thereafter, the length increased again to a certain level below the baseline value plus two standard deviations (SD), at which it seems to stabilize. The difference between means before and during the light stimulation period was found to be significant in all subjects (all p < 0.001). In all subjects, the first few measurements of the series show a declining trend. Since this trend is exactly opposite to the observation during light stimulation, we assume this to be due to dark adaptation when looking into the OCT system, as the luminous exposure is lower at this point. The results of the last ten baseline measurements however are usually stable and show little variance (the mean variance of the last ten measurements is 0.44 μm as opposed to 0.64 μm for all 15 baseline measurements), which leads us to believe that otherwise there is only a negligible or no effect of the measurement light from the OCT system on our results. The measurement lights are a recurring event with every measurement, at baseline as well as during light stimulation, and can therefore be considered a constant that does not affect the comparison between the two states.

### Global averaging to increase signal-to-noise ratio

All A-scans of one volume were used to calculate the A-scan average, which delivers information about the average depth-reflectivity of the photoreceptors and RPE in the scanned region. The advantage of this approach is an increased sensitivity allowing for the detection of small distance changes with a standard OCT system which only provides a limited axial resolution. According to the standard error of the mean, averaging of A-scans leads to a gain in signal-to-noise-ratio (SNR) by a factor equal to the square root of the number of A-scans, when assuming independent identically distributed (IID) Gaussian noise^30^. Here we averaged about 100,000 A-scans, leading to a theoretical increase of the SNR by a factor 100. Zero padding of the data increases the pixel-resolution in depth. Even though lateral information is lost, sensitivity to global axial changes is increased. Scanning over a defined area and averaging the acquired A-scans of locally different positions suppresses spatially limited changes that might not be connected to the stimulus. The use of the position instead of the phase signal has the advantage that the measurements are not dependent on phase stability which requires a very high acquisition speed compared to the bulk motion. These speeds are currently not available in commercial OCT systems. For example, Hillmann *et al*. used a full field OCT system with a maximum A-scan rate of 46.5 MHz, distinctly faster than the Cirrus 4000 HD OCT system employed in our study with an A-scan rate of 27 kHz.

### Physiological considerations in the context of existing work

Both the subretinal space and the distribution of the interphotoreceptor matrix are known to be light sensitive^26,31^. Light has been reported to lead to an expansion of the subretinal space and a decrease of ion concentration in some vertebrate retinas *ex vivo*^26,32^. Water influx, possibly related to RPE cell volume regulation, is believed to be the origin of these effects^33^. The highly specialized interphotoreceptor matrix could likely play a role in the light related changes of the subretinal space. Another process involving the subretinal space is the phagocytosis of shedded discs by RPE cells. Here, cone outer segment shortening was reported^34^. Further, continuous bright light is known to induce massive translocation of phototransduction proteins between the inner and outer rod segments. These processes of adaptation will only occur if a certain threshold of luminous exposure is reached and lasts over a span of several minutes^35^. However, it is not clear if the translocation of this protein could lead to the observed path length change. Furthermore, the exact origin of the RPE reflective band is unknown. If it were mainly formed by scattering from the pigment granules, a migration from the base of the RPE cells into their apical projections as found in retinomotor processes in the guinea pig^36^ would cause a shift of the reflective band towards the outer segment tips. However, no such migration has yet been reported in human subjects.

Three studies using a short light stimulus reported an increase in optical path length between the IS/OS junction and the outer segment tips, the RPE, or both, in mice^17^ and humans^10,11^. Zhang *et al*. showed that this change is not observable if the phototransduction cascade is interrupted early on in mice lacking transducin α^17^. Lu *et al*. reported smaller path length increases for the outer segment’s length than for the IS/OS junction to RPE distance^11^. Hillmann *et al*. reported a length increase of the cone outer segments in the range of nanometers over a timespan of milliseconds to seconds by measuring phase changes^10^. Interestingly, other studies using flashes found effects that reached their maximum much longer after the stimulus compared to this study^11,17^. Furthermore, Hillmann *et al*. were the only ones to reconstruct a clear 2D stimulation pattern from the observed signal^10^. In case of subretinal space changes, this would likely not be feasible, because fluid dynamics of the subretinal space are much less constricted in their expansion, since there are no cell borders in the longitudinal direction. True outer segment elongation, on the other hand, could appear more localized.

According to Rushton’s paradox, a short flash induces an equivalent dark adaption process after exposure as a quantumequivalent 30 second bleach^37^. This indicates that the small differences in stimulation time in the studies using short flashes are not essential to understand the underlying effects. However, studies using minute long stimulation times differ in their evoked responses. A short light flash might not be sufficient to induce some adaptational mechanisms such as the protein translocations mentioned above.

Four other studies, including ours, used long light stimulation^4,18,19^. Investigating light adaptation in mice, it was shown that, contrary to the dark adapted retina, after long light exposure, the reflective bands corresponding to outer segment tips and the RPE become clearly separable by the appearance of a hyporeflective band in OCT images^18,19^. A study by Abrámoff and colleagues using long stimulation times reported IS/OS junction to RPE optical path length decrease in the fovea of healthy human subjects^4^. This is consistent with our findings in the temporal human retina. The composition of the interphotoreceptor matrix varies depending whether the surrounded photoreceptor is a cone or a rod^22^. Given that cones are much more frequent in humans than in rodents, this could be one explanation for the differences in subretinal space changes in response to light between the species.

Our results shown in Fig. 2 reveal that the observed changes in optical path length between IS/OS junction and RPE cannot be assumed to be equal to changes in photoreceptor outer segment length, as is often the case. Here, the major decrease in optical path length (between the IS/OS and the RPE) corresponds to a motion or shift of the RPE towards the outer segment tips and IS/OS junction that is independent of change in the outer segments themselves. Therefore, RPE and photoreceptor outer segment tips cannot be used interchangeably.

### Potential fields of applications

We performed measurements in the temporal human retina at 10 to 20 degrees from the fovea. This more peripheral area was selected for investigation as it contains a large number of rods, the first to be affected in AMD^38,39^. Delayed dark adaptation in rods was recently found to be a biomarker for the early diagnosis of AMD^40^, making a location with high rod content an advantageous area for investigation of this highly prevalent disease. The simplified approach used in this study, combining a dedicated experimental protocol with a standard OCT platform, facilitates easier implementation into clinical routine examinations.

## Conclusion

A new approach to perform optophysiology using a non-dedicated OCT system was presented. Changes in the subretinal space that are representative of adaptational processes can be observed with OCT after light stimulation. To our best knowledge, these effects are reported in the temporal human retina for the first time. Since the introduced method does not require any sophisticated experimental setup, it can be made easily available to researchers and clinicians for intrinsic optical signal imaging. Thus, it might have great potential to serve as an easily implementable tool for the early diagnosis of AMD and other retinal diseases compromising adaptation.

## Materials and Methods

### White light stimulation

**Figure 4:**
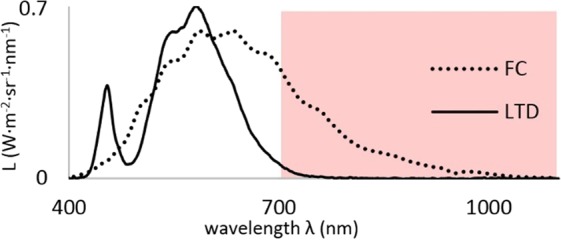
The spectral radiance (L) of the emitted light of both light sources is shown. The infrared component is indicated by the superimposed red rectangle.

A light therapy device for seasonal affective disorder (EnergyLight, Philips International B.V., Amsterdam, The Netherlands) and a fundus camera (FC; FF450 Carl Zeiss Meditec AG, Jena, Germany) were used to deliver white light onto the retina. Both devices differed in radiance (Fig. 4) but delivered light of the same luminance to the retina assuring that the amount of bleached rhodopsin is equal in both experiments (Table 2). The safety limits for exposure of the retina to white light set by the DIN EN ISO guidelines^41^ were not reached or exceeded in this study.

**Table 2:** Benchmarking data of the light stimulation devices.

Device	LTD	FC
Radiance (W ∙ m^−2^ ∙ sr^−1^)	94	155
Luminance (cd ∙ m^−2^)	35 ∙ 10^3^	35 ∙ 10^3^
Infrared component (%)	4	28
Blue component (%)	14	6

The light therapy device (LTD) delivers the same luminance as the fundus camera (FC) at a lower radiance due to its lower infrared contribution.

### Optical coherence tomography

Measurements were performed with a commercially available Cirrus HD-OCT system (Carl Zeiss Meditec, Inc., Dublin, California, USA). The software of the device provided access to raw spectral data and allowed lateral tracking that paused measurements in case of saccades or lateral eye movements. The OCT system operates at a central wavelength of 840 nm and has axial and lateral resolutions of approximately 5 μm and 15 μm in tissue, respectively. The acquisition rate is 27,000 A-scans per second. Fundus imaging is achieved by a scanning laser ophthalmoscope (SLO) channel with a field of view of 36 degrees × 30 degrees. The superluminescent diode (SLD) of the SLO operates at 750 nm, which is dimly visible to the eye. To ascertain that the measurement light from the OCT system did not stimulate the retina we performed 15 baseline measurements to show the variance of measurements in ambient light conditions.

### Measurement protocol

**Figure 5:**
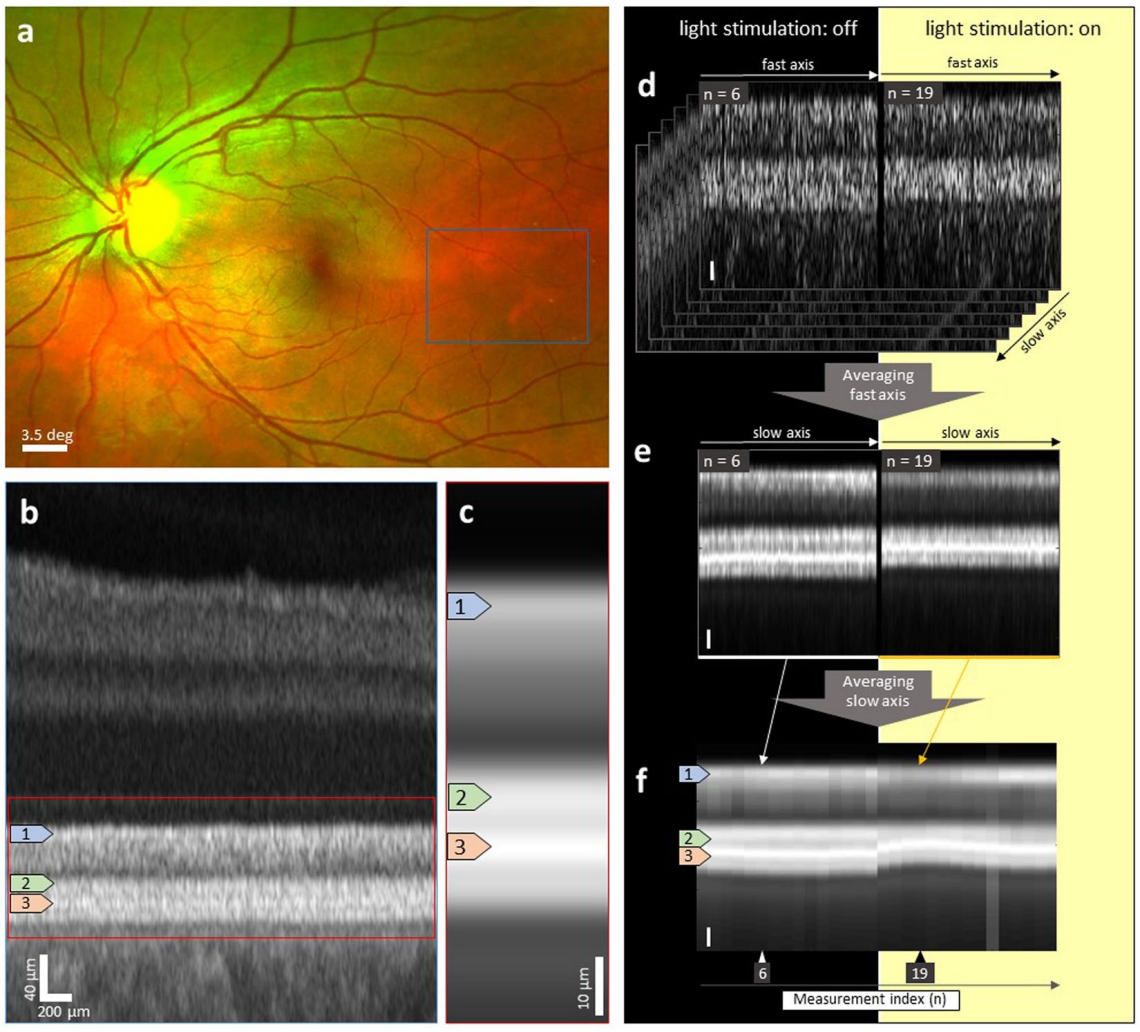
Measurement scheme for assessment of adaptational processes in the peripheral retina. (**a**) Exemplary, selected region of interest in a fundus image (Optomap, Optos plc, Dunfermline, Scotland, United Kingdom). The fundus image is captured using two different wavelengths explaining the colours seen in the figure. (**b**) B-scan in the centre of the region of interest with marked area of investigation in the region of the photoreceptor outer segments. The A-scans were registered to the IS/OS junction and the B-scan was flattened. (**c**) A-scan average - Average of all registered A-scans contained in a singular volumetric dataset of the region of interest. (**d–f**) Measurements with varying degree of averaging before (left) and during (right) the light stimulation period. Scale bars correspond to 10 μm. n is the measurement number and therefore provides a non-linear axis of time. (**d**) Flattened, native B-scans in which each column is one A-scan. (**e**) Each tomogram was averaged along the fast axis and represents one volume. Each column in an image represents one B-scan. (**f**) After averaging all A-scans of one volume (= A-scan average at one time point), the graph shows the optical path length change between IS/OS junction and RPE over time. Each image column represents one volume. A distinct change in optical path length after light exposure is visible. The attribution of the reflective layers in the tomograms is as follows: The first reflective layer after the external limiting membrane (ELM): IS/OS junction (**b–f**:1); second reflective layer: the rods outer segment tips (**b–f**:2) and third reflective layer: RPE (**b–f**:3).

The study was approved by the local ethics board (Ethikkommission der Medizinischen Universität Wien) and the competent authorities and adhered to the guidelines set forth in the Declaration of Helsinki. Study participants gave their written informed consent after explanation of the rational of the study. Five young healthy subjects were recruited and underwent a general medical examination and standard eye examination including perimetry. The study eye received one drop of Mydriaticum 0.5% (AGEPHA Pharma s.r.o., Senec, Slovakia) 20 minutes prior to the measurements to obtain proper pupil dilation. The region of interest was selected 10 to 20 degrees temporally from the fovea to encompass a high number of rods (Fig. 5a) and covered an area of approximately 3.0 × 2.4 mm² in the retina. The measurement region was selected such that the area occupied by vessels was minimized. The entrance position of the measurement beam was chosen to be slightly nasal to the pupil centre to achieve an orthogonal angle of incidence of the probing light onto the retina. Fifteen baseline measurements in ambient light and fifteen measurements after light stimulation were performed under identical experimental conditions. In order to ensure the comparability of the data obtained at different time points and in different illumination conditions, several steps were taken. Baseline measurements were performed under the same experimental conditions as light exposure measurements. Between measurements, the head was removed from and repositioned in the headrest during both, baseline and light stimulation measurements, to produce the same possible variance due to small angular changes and slight position shifts and to investigate the influence of the measurement light of the OCT system on the retina. Using the iris view of the OCT system and repositioning the beam entrance before each measurement, we observed lateral changes that would lead to a maximum alteration of the incidence angle onto the retina in the range of 0.3 degrees. These angular changes would, in theory, lead to an increase of layer distance of 0.00001% in a simplified spherical model. This corresponds well to our preliminary experimental data. When keeping the entrance position of the beam within a range of 150 microns in the pupil plane, we did not detect any changes in layer thicknesses. Tracking software assured lateral stabilization of the measurement. Retrospectively, the success of this lateral stabilization was confirmed by the monitoring of small vessels and other landmarks in *en the face* projections of the measurements (Fig. 1d). The head of the subject was stabilized using a head rest and the line of sight was set using a fixation target. To avoid changes in the angle between the probe beam and retina, the entry position of the beam through the pupil was controlled by adhering to a standardized beam alignment procedure before each measurement. These measures were taken to minimize the variation of beam angles and, more importantly, to ensure that beam angles were as similar as possible for both the baseline and the light exposure series. For light stimulation measurements, subjects were asked to turn their head, position it in the headrest of the light source and abduct the eye under investigation and focus on an external fixation target so that the region of interest was subjected to light. For the first measurement after baseline, the stimulation was performed for five minutes to produce a sufficient adaptation to light and subsequently for one minute between single measurements.

### Nomenclature of retinal layers

There is some controversy about the anatomical correlates to the reflective bands found in OCT tomograms of the outer retina. Jonnal *et al*. pointed out that the use of the term ellipsoid zone^25,42^ was not necessarily more correct than the use of IS/OS junction referring to the second-inner band in OCT data^43^. In order to allow for easy comparison of our findings with data from previous studies related to retinal intrinsic optical signals imaging^10,15,17^, we used the typical nomenclature for the reflective bands found in OCT images of the retina^44,45^. Consequently, we attributed the first band after the ELM to the IS/OS junction and the second to the ROST. A band corresponding to the cone outer segment tips, between IS/OS junction and ROST, was not clearly visible in the A-scan averages of our measurements.

### Data processing

Data processing was performed in MATLAB (The Mathworks, Inc., Massachusetts, USA). The subpixel registration for precise determination of the reflective bands in the OCT images works as follows: First, the axial position map of the IS/OS junction was determined using an algorithm allowing for determination of the axial position with subpixel accuracy, while not increasing the spatial resolution. Second, each A-scan was axially shifted to flatten the retina. The Fourier Transform Shift Theorem, which states that a linear phase term in the frequency domain can be transferred to a delay in the time domain and vice versa^46^ was used to achieve subpixel translation ability as shown in prior publications^47,48^. The position of each layer was defined at its peak, avoiding the influence of changes in intensity. All A-scans of each data set were averaged. As stated by the law of large numbers, the *standard error of the mean* decreases with the number of trials, in this case single A-scans. This, in the present case, leads to a higher sensitivity for global temporal changes. Volume motion (VM) scans, as depicted in Fig. 5f, representing the A-scan averages (Fig. 5c) of particular volumes over time are used to show the temporal changes. In Fig. 5, a difference in the position of layers before and after light stimulation can be seen even in the single B-scans (Fig. 5d) and is more easily discernible after the fast axis, which comprises the A-scans generating one B-scan, has been averaged (Fig. 5e).

Data are presented as means ± standard deviation (SD). The mean path lengths before and during the light stimulation period were compared using a paired t-test and the Bonferroni method was used to adjust for multiple testing. A p-value of 0.05 was considered the level of significance.
